# A Modified Tongue Crib Appliance for Correction of Tongue Thrusting

**DOI:** 10.7759/cureus.40518

**Published:** 2023-06-16

**Authors:** Zahid Manzoor, Ashutosh Wadhawan, Shipra Nagar, Ashish Kumar, Mansi Singh

**Affiliations:** 1 Orthodontics and Dentofacial Orthopaedics, Kalka Dental College and Hospital, Meerut, IND

**Keywords:** interceptive orthodontics, bluegrass appliance, tongue crib, oral habits, tongue thrusting

## Abstract

Digit sucking and tongue thrusting are parafunctional habits that are commonly observed and are recognized as major etiological factors in the development of dental malocclusion. Treatment for these oral habits typically involves removal of the etiology, retraining exercises, and use of mechanical restraining appliances. A 13-year-old male patient reported a complaint of forwardly placed upper front teeth. The extraoral examination of the patient showed a convex profile, good facial symmetry, acute nasolabial angle, incompetent lips at rest, and a shallow mentolabial sulcus. Intraoral examination showed class I molar and canine relations on either side, a mild open bite tendency, a midline diastema, proclined upper and lower anteriors, an increased overjet, and tongue thrusting habit. A modification to the tongue crib was made by adding an acrylic bead in the center of the crib. The patient was trained to roll the bead posteriorly with the aim of retraining the tongue to posture away from the front teeth. Harmful forces of the tongue can result in excessive posterior teeth eruption, open bite, and increased overjet. Duration of appliance wear and type of appliance used are important considerations in treating patients with tongue thrust or open bite. A modified tongue crib was used for six months resulting in significant improvement in maxillary anterior dentition position, lip competence, arch forms, overbite, and midline diastema closure. A tongue crib is a useful tool for addressing tongue thrusting and digit-sucking behaviors by retraining the related muscles, providing physical restraint, and serving as a reminder to break the habit. It can be used in conjunction with a fixed appliance to improve its effectiveness.

## Introduction

Digit sucking and tongue thrusting are the most commonly seen parafunctional habits. These habits are recognized as major etiological factors in the development of dental malocclusion. The relationship between the form and function of the stomatognathic system has been the subject of evaluation by numerous investigators. The proposition that the size [1-5], function [6-8], and posture [9-11] of the tongue may impact the surrounding oral environment has been put forth. However, the question of whether tongue function leads to malocclusion or whether it merely adjusts local occlusion changes has long been a topic of debate [2]. Although certain researchers posit that the size and malfunction of the tongue are fundamental etiological factors in malocclusion development, others contend that tongue thrust swallowing ought to be regarded as a result, rather than the cause, of malocclusion [11-14]. Their reasoning is that in the presence of an overjet or open bite, the act of sealing off the front of the mouth during swallowing becomes difficult. The line of treatment for these habits includes removal of the etiology, retraining exercises, and use of mechanical restraining appliances.

## Case presentation

**Figure 1:**
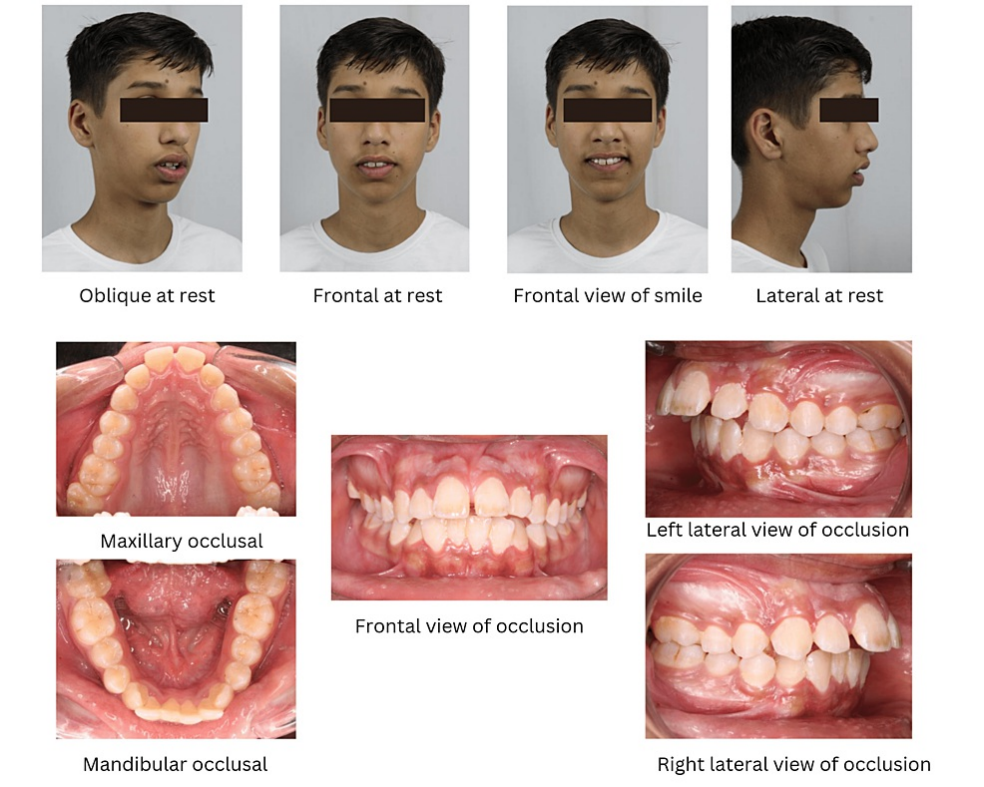
Pre-treatment photographs

**Figure 2:**
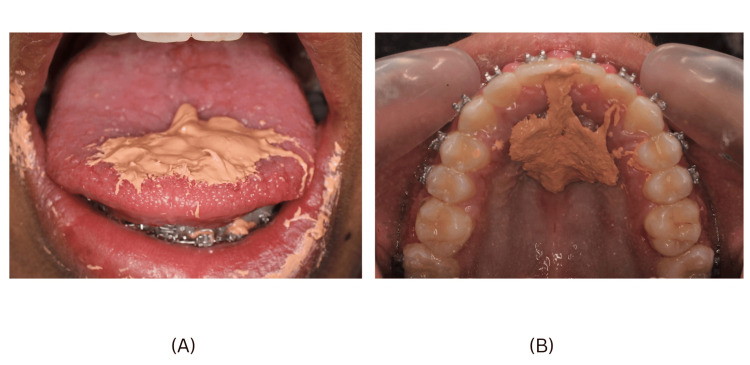
Palatography (A) Impression material loaded onto the tongue. (B) Impression material smeared on palate and maxillary anteriors.

A 13-year-old male patient reported a complaint of forwardly placed upper front teeth. The extraoral examination of the patient showed a convex profile, good facial symmetry, acute nasolabial angle, incompetent lips at rest, and a shallow mentolabial sulcus. Intraoral examination showed class I molar and canine relations on either side, a mild open bite tendency, tongue thrusting habit, a midline diastema, proclined upper and lower anteriors, and an increased overjet along with a predominantly oral mode of respiration (Figure 1). A detailed history of the patient's tongue habit was elicited, during which the patient reported placing their tongue forward against the anterior teeth while swallowing. Palatography and cineradiography are two methods employed to investigate and evaluate swallowing patterns. Palatographic analysis (Figure 2) was done to confirm the tongue thrust swallow pattern wherein impression material was loaded onto the tongue and the patient was instructed to swallow. Impression material was smeared on the palate towards the anterior teeth suggesting a forward position of the tongue on swallowing. On the basis of clinical investigations, palatography, and patient history, a diagnosis of tongue thrusting with mouth breathing was made.

Appliance design

The bluegrass appliance is indicated for those children who have continued thumb-sucking habit that is affecting their mixed or permanent dentition.

A tongue crib is a habit-breaking appliance effective in breaking the tongue habit of thrusting. It has been suggested by Fink [15] that the effectiveness of crib therapy is more due to a redirected resting position of the tongue than a dynamic restraint of the tongue.

**Figure 3:**
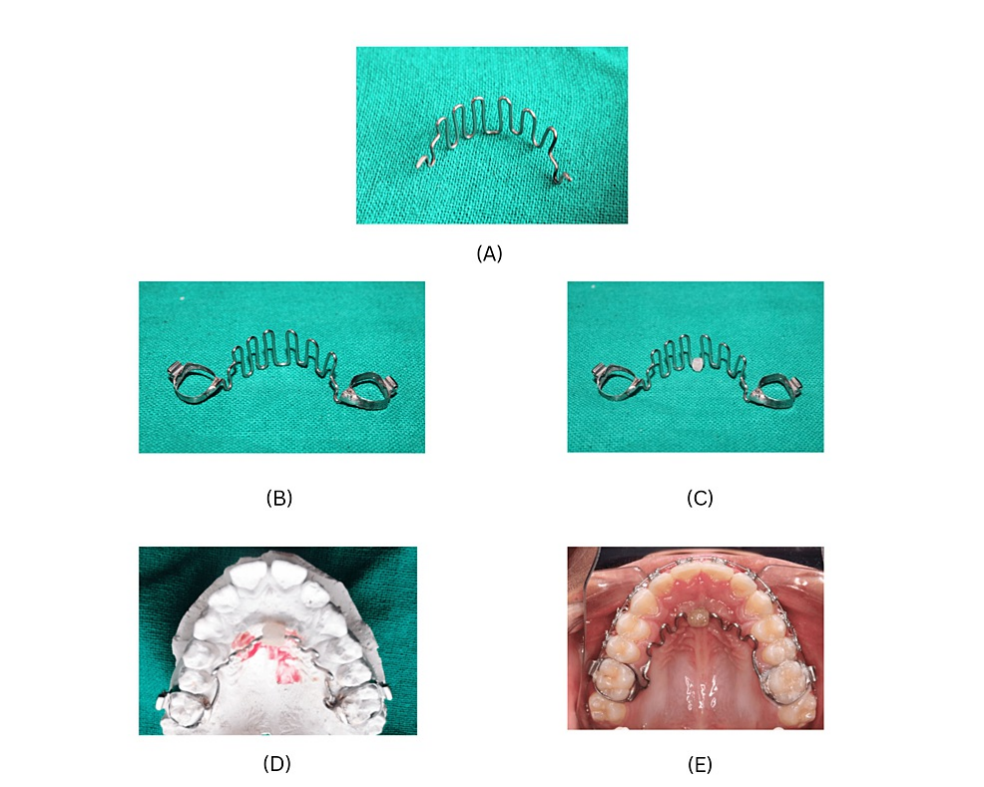
Appliance design (A) Tongue crib fabricated in 0.045" SS wire. (B) Tongue crib soldered onto molar bands. (C) Acrylic bead added to tongue crib. (D) Appliance adapted to patient's cast. (E) Appliance fixed intraorally.

A palatal crib was fabricated in a 0.045" steel wire (Figure 3A) and soldered onto molar bands (Figure 3B). A modification to the tongue crib was made by adding an acrylic bead in the center of the crib (Figure 3C). The patient was trained to roll the bead posteriorly with the aim of retraining the tongue to posture away from the front teeth.

The modified tongue crib differs from the conventional crib in providing a dual advantage of physical restraint of the tongue along with positive reinforcement and retraining the tongue from the acrylic roller.

A similar appliance was utilized by Abraham et al. [16] in a patient with tongue thrust. They noticed the achievement of somatic swallow post-habit-breaking therapy.

Other appliances used in the correction of tongue thrust habit are tongue rakes, bonded tongue spurs, and the blue grass appliance.

## Discussion

The presumed correlation between the movements of the tongue, lips, and mandible during the act of swallowing and the morphology of the contiguous hard structures has engendered the concepts of "normal" and "abnormal" swallowing. The causative factors contributing to the protrusion of the tongue tip during swallowing encompass oropharyngeal development and maturation, oral habits, and environmental influences [17].

According to Hanson et al. [18] excessive posterior teeth eruption, open bite, and/or increased overjet result due to the harmful forces of the tongue.

A cinefluorographic study conducted by Cleall [19] examined tongue movement in patients with a palatal crib. The findings revealed that the use of the crib caused the tongue to function in a higher and more posterior position. In another investigation, Sayin et al. [20] utilized magnetic resonance imaging (MRI) to study the initial effects of the tongue crib and observed that the tongue tip was positioned further posteriorly when the appliance was worn, which necessitated adaptive changes.

However, according to Subtelny and Sakuda [21], the effectiveness of crib therapy was limited in patients who wore the appliance for less than six months. To address this limitation, research has indicated that a fixed appliance that does not require compliance may lead to more favorable outcomes in the correction of open bite and the inhibition of habits. Therefore, clinicians must take into account the duration of appliance wear and the type of appliance used when treating patients with tongue thrust or open bite.

According to Proffit [22], the duration of light pressure resulting from the tongue adopting a forward resting posture has the potential to exert a more substantial influence on tooth positioning, both vertically and horizontally, in comparison to the relatively short-lived impact of a tongue thrust swallow.

An alternative respiratory pattern, wherein an individual breathes through the mouth instead of the nose, has the capacity to bring about postural adjustments in the head, jaw, and tongue. In order to respire through the mouth, it is necessary to lower the mandible and tongue while simultaneously extending (tilting back) the head [22].

**Figure 4:**
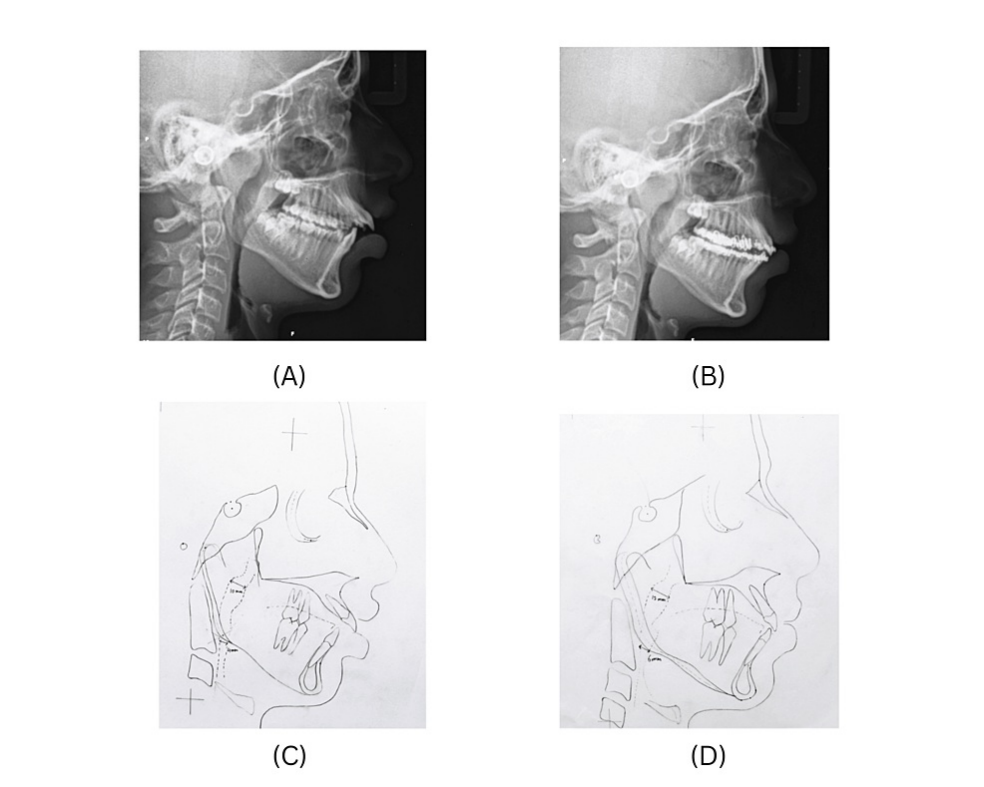
Airway analysis (A) Pre-treatment lateral cephalogram. (B) Present stage lateral cephalogram. (C) Pre-treatment airway analysis: Upper pharyngeal airway: 10 mm Lower pharyngeal airway: 4 mm. (D) Present stage airway analysis: Upper pharyngeal airway: 12mm Lower pharyngeal airway: 6 mm.

Cephalometric analysis of the airway revealed reduced upper and lower pharyngeal airway dimensions (Figure 4C). Reduced airways can predispose normal children to anterior tongue positioning. A pre- and present-stage comparison of the airway revealed an improvement in the airway dimensions (Figure 4).

**Figure 5:**
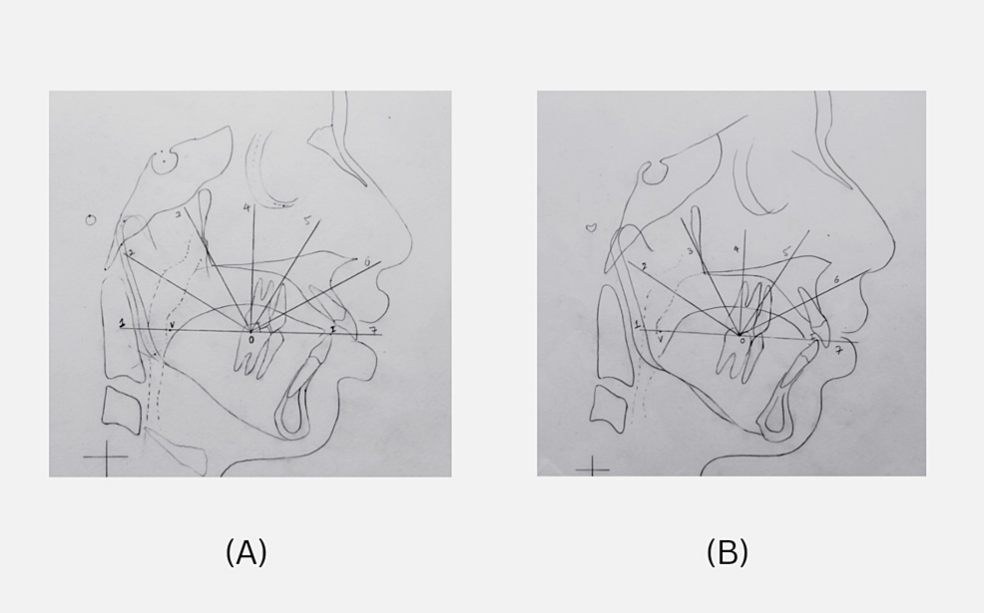
Analysis of tongue position by cephalometric radiography Cephalometric analysis of tongue [23]. (A) Pre-treatment values: Position 1 - 4 mm, position 2 - 10 mm, position 3 - 10 mm, position 4 - 12 mm, position 5 - 12 mm, position 6 - 10 mm, position 7 - 9 mm. (B) Present stage values: Position 1 - 6 mm, position 2 - 12 mm, position 3 - 13 mm, position 4 - 12 mm , position 5 - 12 mm, position 6 - 6 mm, position 7 - 7 mm.

Pre-treatment cephalometric analysis of the tongue (Figure 5) suggested a lower position of the tongue in relation to the palate (Figure 5A). An improvement in the position of the tongue was noticed during post-appliance therapy as indicated by the present stage values (Figure 5B).

A modified tongue crib (Figure 3) was utilized for a period of six months during which the patient was motivated to roll the acrylic ball posteriorly in an attempt to improve the tongue posture. Significant improvement was seen in the position of the maxillary anterior dentition which is reflected in the cephalometric values (Table 1). Retention protocol involves follow-up for six months post-appliance removal.

**Table 1:** Cephalometric values

Sl. no	Cephalometric parameter	Mean value	Pre-treatment value	Present stage value
1.	UI/NA	22°/4mm	42°/10 mm	26°/5mm
2.	LI/NB	25°/4mm	29°/8 mm	31°/9mm
3.	Inter incisal angle	131+/-5°	104°	117°
4.	IMPA	90°	92°	90°
5.	U1 to palatal plane	30.5 +/-2.1 mm	29 mm	30 mm
6.	U6 to palatal plane	26.2 +/- 2.2 mm	23 mm	24 mm
7.	Intercanine width		Upper - 32mm; Lower - 26 mm	Upper - 35 mm; Lower - 28 mm

**Figure 6:**
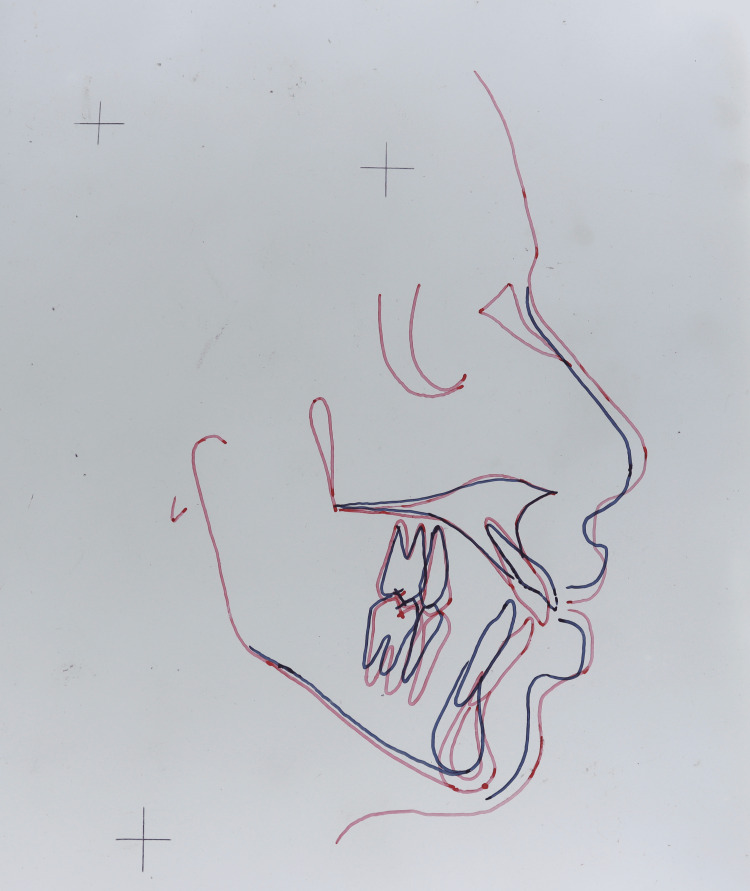
Cephalometric superimposition

A marked improvement in lip competence at rest can be seen in cephalometric superimposition (Figure 6).

**Figure 7:**
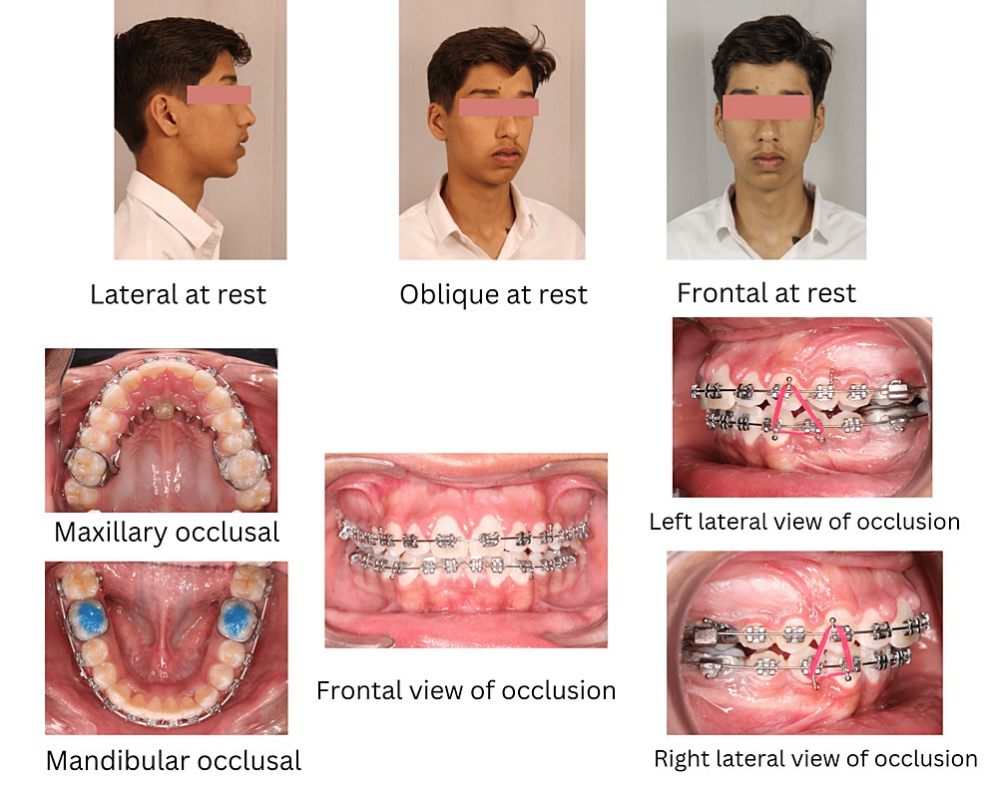
Photographs of the present stage

The combination of habit-breaking appliances along with fixed mechanotherapy also resulted in improved arch forms, positive overbite, and spontaneous closure of midline diastema (Figure 7).

## Conclusions

The modified tongue crib is a useful tool for addressing tongue thrusting and digit-sucking behaviors by retraining the related muscles, providing physical restraint, and serving as a reminder to break the habit. It can be used in conjunction with a fixed appliance to improve its effectiveness. Additionally, it can be utilized to correct posterior crossbites.

## References

[1] Hotokezaka H, Matsuo T, Nakagawa M, Mizuno A, Kobayashi K (2001). Severe dental open bite malocclusion with tongue reduction after orthodontic treatment. Angle Orthod.

[2] Liu ZJ, Shcherbatyy V, Gu G, Perkins JA (2008). Effects of tongue volume reduction on craniofacial growth: a longitudinal study on orofacial skeletons and dental arches. Arch Oral Biol.

[3] Lowe AA, Takada K, Yamagata Y, Sakuda M (1985). Dentoskeletal and tongue soft-tissue correlates: a cephalometric analysis of rest position. Am J Orthod.

[4] Turner S, Nattrass C, Sandy JR (1997). The role of soft tissues in the aetiology of malocclusion. Dent Update.

[5] Vig PS, Cohen AM (1974). The size of the tongue and the intermaxillary space. Angle Orthod.

[6] Alexander S, Sudha P (1997). Genioglossis muscle electrical activity and associated arch dimensional changes in simple tongue thrust swallow pattern. J Clin Pediatr Dent.

[7] Fuhrmann RA, Diedrich PR (1994). B-mode ultrasound scanning of the tongue during swallowing. Dentomaxillofac Radiol.

[8] Lowe AA (1980). Correlations between orofacial muscle activity and craniofacial morphology in a sample of control and anterior open-bite subjects. Am J Orthod.

[9] Karacay S, Akin E, Ortakoglu K, Bengi AO (2006). Dynamic MRI evaluation of tongue posture and deglutitive movements in a surgically corrected open bite. Angle Orthod.

[10] Proffit WR, Fields HW, Sarver DM, Ackerman JL (2000). Contemporary Orthodontics. Proffit WR. Contemporary Orthodontics, 3rd ed. St Louis: Mosby; 2000:134-8..

[11] Proffit WR (1978). Equilibrium theory revisited: factors influencing position of the teeth. Angle Orthod.

[12] Hanson ML (1988). Orofacial myofunctional therapy: historical and philosophical considerations. Int J Orofacial Myology.

[13] Lauder R, Muhl ZF (1991). Estimation of tongue volume from magnetic resonance imaging. Angle Orthod.

[14] Schwestka-Polly R, Engelke W, Hoch G (1995). Electromagnetic articulography as a method for detecting the influence of spikes on tongue movement. Eur J Orthod.

[15] Fink FS (1990). Resting position of tongue important. Angle Orthod.

[16] Abraham R, Kamath G, Sodhi JS, Sodhi S, Rita C, Sai Kalyan S (2013). Habit breaking appliance for multiple corrections. Case Rep Dent.

[17] Subtelny JD, Subtelny JD (1973). Oral habits - studies in form, function, and therapy. Angle Orthod.

[18] Hanson ML, Andrianopoulos MV (1982). Tongue thrust and malocclusion: a longitudinal study. Int J Orthod.

[19] Cleall JF (1965). Deglutition: a study of form and function. Am J Orthod.

[20] Sayin MO, Akin E, Karaçay S, Bulakbaşi N (2006). Initial effects of the tongue crib on tongue movements during deglutition: a cine-magnetic resonance imaging study. Angle Orthod.

[21] Subtelny JD, Sakuda M (1964). Open bite: diagnosis and treatment. Am J Orthod.

[22] Proffit WR, Fields HW, Larson B, Sarver DM (2018). Contemporary Orthodontics. https://books.google.co.in/books?hl=en&lr=&id=A45nDwAAQBAJ&oi=fnd&pg=PP1&dq=proffit+contemporary+orthodontics&ots=-wMmbSURTj&sig=QRPGSaR4ni6M73iRW1AIvWy0uUA&redir_esc=y#v=onepage&q=proffit%20contemporary%20orthodontics&f=false.

[23] Rakosi T (1982). An atlas and Manual of Cephalometric Radiography. https://www.academia.edu/25983047/An_Atlas_and_Manual_of_Cef_alometric_Radiography.

